# Culturally-Tailored Smoking Cessation for American Indians: Study protocol for a randomized controlled trial

**DOI:** 10.1186/1745-6215-12-126

**Published:** 2011-05-18

**Authors:** Won S Choi, Babalola Faseru, Laura A Beebe, Allen K Greiner, Hung-Wen Yeh, Theresa I Shireman, Myrietta Talawyma, Lance Cully, Baljit Kaur, Christine M Daley

**Affiliations:** 1 Department of Preventive Medicine and Public Health, University of Kansas Medical Center, Kansas City, KS, USA; 2 Center for American Indian Community Health, University of Kansas Medical Center, Kansas City, KS, USA; 3 Department of Family Medicine, University of Kansas Medical Center, Kansas City, KS, USA; 4 Department of Biostatistics, University of Kansas Medical Center, Kansas City, KS, USA; 5 University of Oklahoma Health Science Center, Oklahoma City, OK, USA

## Abstract

**Background:**

Cigarette smoking is the number one cause of preventable death among American Indian and Alaska Natives, AI/ANs. Two out of every five AI/AN will die from tobacco-related diseases if the current smoking rates of AI/ANs (40.8%) persist. Currently, there is no proven, effective culturally-tailored smoking cessation program designed specifically for a heterogeneous population of AI.

The primary aim of this group randomized clinical trial is to test the efficacy of "All Nations Breath of Life" (ANBL) program compared to a non-tailored "Current Best Practices" smoking cessation program among AI smokers.

**Methods:**

We will randomize 56 groups (8 smokers per group) to the tailored program or non-tailored program for a total sample size of 448 American Indian smokers. All participants in the proposed study will be offered pharmacotherapy, regardless of group assignment. This study is the first controlled trial to examine the efficacy of a culturally-tailored smoking cessation program for American Indians. If the intervention is successful, the potential health impact is significant because the prevalence of smoking is the highest in this population.

**Trial Registration:**

ClinicalTrials.gov: NCT01106456

## Background

Although American Indian and Alaska Natives (AI/ANs) make up approximately 1% of the US population, they have the highest smoking rates of any racial/ethnic group in the US [1]. Smoking rates among AI/AN smokers vary by region and are highest in the Northern Plains 44.1%, and lowest in the Southwest 21.2% [2,3]. AI/AN smokers have more difficulty quitting smoking compared to other ethnic groups, [4] evidenced by their significantly lower quit ratios [5,6], and are among the least successful in maintaining long term abstinence.

Cigarette smoking is the number one cause of preventable death among AI/ANs [7,8]. The death rate among AI/ANs due to tobacco use is double that of other groups in the United States [8].

There are currently no proven, effective smoking cessation programs designed specifically for a heterogeneous population of AI/AN. A few attempts at the tribal level have proven effective and a few untested attempts have been made for diverse groups. The "It's Your Life - It's Our Future" project in Northern California used messages related to cultural identity, responsibility to family and tribe, and respect for tobacco products [6]. The program was tailored to the California AI/AN community and had a 5.7% quit rate at 18-month follow-up for the intervention group versus a 3.1% quit rate for the control group. The Giving American Indians No-smoking Strategies (GAINS) study was conducted to determine the feasibility and effectiveness of delivering a smoking cessation intervention through health clinics serving urban AI/ANs sites in Seattle, Milwaukee, Minneapolis, and Spokane [9]. A major goal was to implement a culturally appropriate adaptation of the Doctors Helping Smokers (DHS) model [10]. The GAINS intervention incorporated five major principles: (1) screening of patients for smoking status, (2) use of smoke card as a reminder to providers, (3) discussion of smoking cessation with a clinician, (4) supportive reinforcement by clinic staff, and (5) monitoring of quit progress using the smoke cards. The 7-day point prevalence abstinence rates were not different between the intervention group (6.7%) and the control group (6.8%) at one year follow-up. Potential reasons for this null effect include: incomplete implementation, large proportion of subjects were not exposed to the intervention, early problems with delivery of the program by clinic staff, high non-response rates, and a need for further cultural-targeting. No formative research was conducted to determine acceptability of the intervention to the target population.

The "Second Wind" smoking cessation curriculum (developed by the Muscogee Nation in Oklahoma) is based on the FreshStart curriculum created by the American Cancer Society. It consists of six group sessions, with groups meeting every two weeks for counseling and receipt of pharmacotherapy. Second Wind utilizes the "Talking Circle" format, which is a familiar, comfortable, and culturally acceptable tradition within many AI/AN communities [11]. Historically, talking circles have been used by many tribes as a vehicle for discussing and presenting important issues relevant to all members of the tribe. Although this program was recently developed and disseminated to several tribes, no data on its efficacy are available because no formal evaluation to determine its impact on smoking cessation was conducted. In summary, there is a lack of empirically validated programs for smoking cessation in the AI/AN population and therefore, an urgent need for programs such as the ANBL.

Current treatment guidelines recommend that behavioral counseling and pharmacotherapy be offered to any smoker interested in quitting [12]. A strong dose-response relationship has been found between the amount of personal contact with program staff and smoking cessation. Four or more sessions are recommended. Treatment by nurses, behavioral counselors, physicians, and other clinician types are all effective. Pharmacotherapy doubles quit rates when compared to placebo [13], and the highest abstinence rates are achieved when pharmacotherapy is combined with counseling [14]. Our intervention provides pharmacotherapy to all participants and targeted counseling delivered by AI counselors who have worked closely with the American Indian communities and respect the cultures, values, and traditions of the Indian people. Therefore, our intervention includes the current "best practice" recommendations for smoking cessation as well as a culturally-tailored group counseling program.

There is much diversity in tobacco use among AI/ANs throughout the country. Along with such plants as sage, cedar, sweet grass, and red willow bark, tobacco has long been a sacred plant for many AI/AN tribes [15]. Because tobacco was not native to all areas of the United States, a number of AI/AN tribes did not use it historically for ceremonial or spiritual purposes. However, early use of tobacco in smoke offerings, pipe smoking, burial services, gift-giving, and for medicinal purposes is well-documented, particularly among tribes native to the eastern seaboard and the Great Plains (e.g. Six Nations of the Iroquois, [16] Delaware, Nanticoke, [17] Lakota [18]). Many AI/AN people believe that tobacco connects them to the spirit world through prayer (e.g. Lenape) [19,20]. Tobacco is also used at AI/AN ceremonies, such as a sweat lodge, Sun Dance, or *Yuwipi *(e.g. Lakota) [21]. It is considered a gift from the Creator (e.g. Absarokas and Hidatsa) [20]. Traditional tobacco use is common in the AI/AN population and has diffused to many other AI/ANs who did not historically use it, particularly at powwows, where it is often given as a gift to the host drum [22]. When developing a smoking cessation program for a heterogeneous population, such as the one in Kansas where over 200 different groups are represented, it is imperative to acknowledge the diversity of traditional ritual and spiritual use of tobacco. It is also important to differentiate between traditional tobacco use and recreational smoking and acknowledge that conventional tobacco control messages that portray tobacco entirely negatively may be offensive and hence ineffective in AI/AN communities [23].

There is a desperate need for effective, culturally tailored cessation programs [24,25]. The primary aim of this group randomized clinical trial is to test the efficacy of "All Nations Breath of Life" (ANBL) program compared to a non-tailored "Current Best Practices" smoking cessation program among AI smokers.

## Methods/Design

All Nations Breath of Life (ANBL) was developed using the principles of community-based participatory research [26] that include: 1) tailoring to meet the needs of individuals and communities; and 2) providing the opportunity for the people for whom the program is developed to participate in the development, implementation, and evaluation. To address these principles and to ensure that the program meets the needs of those it is designed to help, we involve AI/AN community members at all points of program development, allowing us to tailor our program to the AI/AN population. We also recognize that our target AI/AN population is not a community defined by geopolitical borders, but by a shared identity. This shared native identity, however, has multiple manifestations that do not form one overarching culture (defined as a system of knowledge, beliefs, values, morals and symbols, in terms of which human groups interact among themselves, with other groups, and with their natural environment) [27]. Ethnic identity can best be understood as a way of defining self from others. In the case of many AI/AN, ethnic identity when talking with a non-Native person is supra-tribal; in other words, it is a shared identity of being AI/AN [28]. Culture is tribe-specific. The AI/AN designation is used to describe a heterogeneous group of people by the U.S. government. Because of this heterogeneity, tailoring interventions to the community or "culture" becomes exceedingly difficult. We address this heterogeneity throughout our program by incorporating ideas and practices from many AI/AN cultures.

In order to implement the ANBL program, we will use AI facilitators for our group sessions. The program includes interactions between individual smokers and the facilitators, as well as group dynamics within each cessation group. Group dynamics involve smokers' interactions with each other, as well as with the facilitator. Our use of the talking circle format, as well as our use of AI/AN facilitators, is designed to facilitate positive group dynamics.

### Design Overview

**Figure 1:**
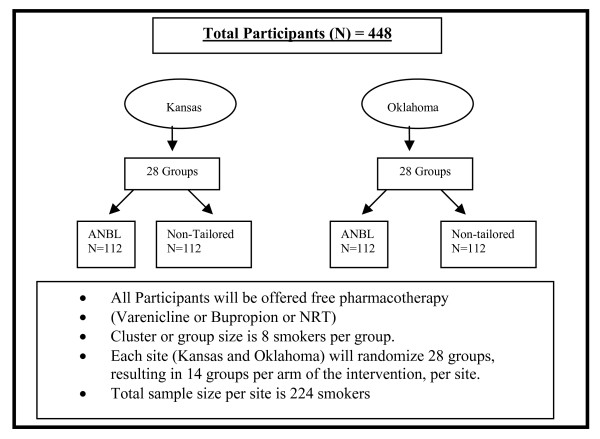
**Design of Randomized Clinical Trial of Smoking Cessation among American Indians**.

Figure 1 provides an overview of the study. This study will use a group randomized multi-site clinical trial design to examine the efficacy of a culturally-tailored smoking cessation program (**ANBL**) for AI/AN smokers versus Non-tailored Current Best Practices (**CBP**). AI/AN smokers in two sites (Kansas and Oklahoma) will be group randomized to either ANBL or Non-tailored (CBP). Each site (KS and OK) will randomize 28 groups, resulting in 14 groups per arm of the intervention. Participants in both groups (ANBL and CBP) will be offered pharmacotherapy (e.g. varenicline or bupropion or NRT). The primary outcome of interest will be biochemically verified continuous abstinence at 1 year. Secondary endpoints include number of quit attempts and number of cigarettes smoked (among continuing smokers), pharmacotherapy utilization, and the number of completed group sessions. We will also examine the marginal cost-effectiveness of the intervention.

The study will proceed in three phases over a five-year period. Phase I will consist of development and training. Phase 2 will be to conduct the randomized trial, and Phase 3 consists of data analysis and dissemination.

Participants will be recruited from two sites: Kansas and Oklahoma. Although the ANBL arm is a group intervention and the CBP arm is individual standard care, the unit of randomization will be at the group level. Recruitment and randomization will be balanced by site: both sites will recruit until we reach our target sample size of 448 AI/AN smokers who are at least 18 years of age (see eligibility criteria in Table 1). Each site will randomize 28 groups (14 groups per arm) with 8 smokers in each group for a total site sample size of 224 smokers per site.

**Table 1:** Eligibility Criteria for Participants

Eligibility criteria	
**Inclusion Criteria**	**Exclusion Criteria**

Age 18 years or older Have a home address and telephone number Willing to participate in all study components Willing to be followed-up for 12 months Smoked at least 100 cigarettes in their lifetime Current smoker American Indian or Alaska Native (self-identified)	Planning to leave the state within next 24 months Pregnant or breast feeding or planning to become pregnant in next 4 months. Medically ineligible after screening

### Randomization Process

All participants who consent to participate will be randomized to either the **ANBL **or **CBP **arm. Randomization will be performed at each site after recruitment, the baseline telephone assessment and determination of eligibility. Participants will form temporal clusters in recruiting order (i.e. the first 8 participants form cluster 1, the second 8 participants form cluster 2, and so on), and then pairs of clusters will be assigned to the groups using randomized permuted blocks based on a computer generated random numbers to guarantee the balance between arms at each site (Kansas and Oklahoma) [29]. For example at each site, the first pair of clusters will be assigned to the ANBL and the CBP groups, respectively, and the second pair of clusters will be assigned to the CBP and the ANBL groups, respectively. Thus, the main purpose of this allocation scheme is to assure balance with respect to the number of participants assigned to each arm of the trial in a logistically convenient fashion. This process continues until we end up with 14 clusters in the ANBL arm and 14 clusters in the CBP arm at each site. Facilitators will be responsible for delivering the interventions and separate assessors will be responsible for conducting assessments.

### All Nations Breath of Life (ANBL) Intervention Components

The current version of ANBL has five primary components, designed by our pilot participants and research team members, These are: (1) group support sessions, (2) individual telephone counseling using Motivational Interviewing (MI), (3) a culturally tailored educational curriculum, (4) pharmacotherapy, and (5) participant incentives, all of which have been tailored specifically to a heterogeneous group of AI/AN people.

#### Group Support Sessions

During our initial discussions with community members about what they would like to see in a smoking cessation program, nearly everyone wanted group support led by a community member. Therefore, the primary component of ANBL is a series of group-based support sessions. We discussed who would lead the sessions at length, particularly whether they needed to be trained counselors or have certain educational degrees. We decided the most important factor when choosing individuals to lead the groups was their ties to the AI/AN community. Due to low rates of educational attainment in the AI/AN community, we decided it would be best to not limit session leaders to individuals with counseling degrees and/or experience. Instead we decided to train community members in group support and counseling skills. We also decided not to call our group leaders "counselors" due to a reticence on the part of community members to trust providers trained in Western counseling techniques. In addition, the term "counselor" implies the group leader is above group members and is the most qualified to give advice. We chose the term "facilitator" because we see this role as facilitating discussion and creating positive group interaction. The group members themselves provide the majority of support and advice, with help from the facilitator and educational materials. We have been lucky to find community members with social work or psychology backgrounds willing to help develop methods for group building and support facilitation. All group sessions begin with team building exercises and personal discussion among members about things they might be experiencing in their lives, both those related directly to smoking and other things that may come up that help or hinder quit attempts. Our first group session is devoted largely to forming a cohesive group and learning all members' personal stories about their life-long journeys with tobacco, both sacred and recreational.

#### Individual Telephone Counseling

Our participants and community partners have stressed the need for some individual counseling, particularly for people who are uncomfortable talking about certain things in our mixed-gender, mixed-age, mixed-tribe groups. Therefore, our most recent incarnation of ANBL includes telephone calls that go beyond a reminder about the next group session (the original purpose of the calls). Between group sessions, facilitators call participants to see how they are doing, provide counseling for personal issues, and to remind them of the next group session. Facilitators also ask about side effects of medication and any adverse events. During these calls, facilitators will use Motivational Interviewing (MI), which has been found to be effective among AI/AN [30]. MI is designed to enhance motivation for change, and is based on the assumption that many individuals with addictions are not in an advanced state of readiness to change. The goal of MI is to increase an individual's motivation to change by developing a discrepancy between current behavior and goals and/or values.

#### Educational Curriculum

Our educational curriculum is divided into 11 brochures, given throughout the program, and combines the latest smoking cessation methods with culturally-specific elements. Community members have stressed throughout development of the program that cultural issues must be ingrained within the program and its accompanying education materials, not given "lip service" by putting pictures of AI/AN people on otherwise "White" materials. Our materials have evolved through several iterations over time [31-34]. Our current materials were created by our research team and Community Advisory Board (CAB) members, with input from pilot participants, and then sent to an AI/AN graphic artist (Nakota Designs, Inc.), for final layout.

### Pharmacotherapy Offered to All Participants

Current treatment guidelines recommend pharmacotherapy be offered to all smokers making a quit attempt. The PI and site PI of this study along with our AI/AN community members agreed that all participants (ANBL and CBP arms) had to be offered pharmacotherapy (varenicline or bupropion or NRT) as a component of the study design. This decision was a collaborative one between the AI/AN tribes and the investigators who have worked with this population. The AI/AN community will not accept placebo treatment, therefore, the interventions would not be acceptable or feasible to potential participants if a placebo was part of the study design. In addition, we believed it was ethically imperative that we provide smoking cessation interventions consistent with the current clinical practice guidelines for the treatment of tobacco use. Consequently, we provide participants with their choice of free pharmacotherapy, including Chantix^®^, Zyban^®^, Nicotine Replacement Therapy (NRT, patches, gum, or lozenges), or a combination of the latter two. Participants can also choose no pharmacotherapy. All facilitators are trained in providing advice and addressing questions related to pharmacotherapy and discuss the options with participants, also asking about medical history to determine if there is any reason the individual should not be taking medication. Participants are also given educational brochures about the options and have the option of talking with a physician privately. All participants requesting prescription medication must be medically cleared prior to receiving medication. Study staff ensure that providers in the area are informed about the study prior to recruitment, and work with participants and providers to ensure that smokers wanting prescription pharmacotherapy receive it expeditiously. As all forms of NRT are over-the-counter medications, participants are not required to obtain physician clearance to take them; however, they receive written information about them and facilitators explain risks.

### Participant Incentives

Incentives were designed by our AI/AN research team members and our CAB. Every week, participants receive a "quit kit", a variety of hard candy, gum, flavored toothpicks, straws, American Indian snacks, etc., to help them avoid smoking. Quit kits have been used successfully in our pilot study, as well as in multiple other smoking cessation programs [29,30]. Participants are given a stress ball as a part of their initial quit kit, as well as a magnet with a hole cut in the center to put a picture. The magnets say "I want to quit smoking for..." and participants can put a picture of themselves or someone they care about. The idea for magnets came from participants who talked about quitting for family members, especially grandchildren. For the session devoted to traditional tobacco use, we originally had tribal elders come in and talk about personal experience. However, our participants felt it would be more appropriate to talk amongst themselves and show a video of elders talking about traditional use in different tribes. We have since filmed several elders and are creating a video to watch during the session and give as an incentive. When discussing stress management techniques, emphasis is placed on traditional activities and participants are led through a stress reduction activity that uses Native flute music. We have worked with two local flute players to purchase copyrights to their music to develop CD's. To complement discussion of weight management, we provide a copy of the DVD Rez-robics^®^, a set of two aerobic videos that incorporate traditional dance. In an earlier version of ANBL, participants were provided with food during three group sessions, healthy snacks for weight maintenance, a meal with a family member or friend for social support, and a celebratory meal at the final regular group session. After discussions with our CAB, we decided that all of these meals/snacks will become potluck, with participants and facilitators bringing in food. This decision was made to help the sustainability of the program in the real world. The fact that KUMC owns the copyright to the DVD and flute music CD adds to our sustainability because the only cost involved in them is $1 per copy. Rez-robics^® ^may be distributed free of charge to any AI/AN person.

At the Week 1 individual session and week 12 group and month 6 group sessions, we provide a $20 gift card for their time. All participants receive the gift cards for their time, regardless of smoking status and willingness to provide saliva for verification. Detailed descriptions of session topics and incentives are shown in Table 2.

**Table 2:** Flow of Participants for ANBL

Week #	Type of Session	Topics Covered	Brochures/Other Handouts	Incentives
Screening	Telephone Intake	Current smoking levels Level of traditional/ceremonial use Readiness to quit	None	None

1	Individual in-person meeting with facilitator	Program start date Quit date information Questions about program Personal history with smoking	NRT Pharmacotherapy Preparing to Quit Cigarette smoking and Native People	Tote bag and Binder for educational materials Photo magnet ANBL pen $20 gift card Pharmacotherapy

2	Group in-person	Personal histories with smoking Team building Social support I Coping with withdrawal	Why people smoke Quit Contract Quit reasons card	Quit kits Pharmacotherapy Water bottles

3	Group in-person	Discussion of current personal issues with quitting or other Facts about smoking	Things instead card	Quit kit refills Pharmacotherapy ANBL bracelet

4	Group in-person	Traditional use of tobacco Discussion of current personal issues with quitting or other	Traditional use of tobacco	Quit kit refills Pharmacotherapy Traditional Use DVD

5	Group in-person	Stress management Discussion of current personal issues with quitting or other	Stress reduction and management American Indian flute music Meditation techniques	Quit kit refills Pharmacotherapy Flute music CD Stress balls

6	Group in-person	Weight management Healthy eating and exercise Discussion of current personal issues with quitting or other Reservation based aerobic exercise techniques	Weight management during smoking cessation Discussion of Healthy American foods (Healthy eating at Powwows)	Quit kit refills Pharmacotherapy Rez-robics DVD or VHS Pedometers Healthy snacks

7	Group in-person	Social support II Discussion of current personal issues with quitting or other	Friends and family and quitting smoking	Quit kit refills Pharmacotherapy Meal with family member/friend

8	Group in-person	Staying quit Discussion of current personal issues with quitting or other	Staying quit	Quit kit refills Pharmacotherapy ANBL T-shirt

9	Telephone MI	Personal issues	None	None

10	Telephone MI	Personal issues	None	None

11	Telephone MI	Personal issues	None	None

12	Group in-person	Celebration Discussion of how traditional worldview and behaviors helped in the quitting process Feedback on program	None	Certificate of Achievement Celebratory potluck meal $20 gift card

1 week prior to 6 months follow-up	Telephone MI	Personal issues Reminder to return to 6 month follow-up	None	None

6-month	Group in-person	Current smoking status Personal issues	None	$20 gift card

12-Month	Individual in-person	Current smoking status	None	$20 gift card

### Session procedures and time commitment

#### Screening: Telephone Intake

The majority of intake interviews is conducted via telephone, but may be done in person at any program site. The initial screening reviews inclusion/exclusion criteria, personal smoking levels and readiness to quit, and basic information about the program. We estimate intake to take 10-15 minutes. It is done by research assistants for each program site or facilitators.

#### Individual In-person Meeting with Facilitator

Potential participants meet with their group facilitator individually prior to the first group session. Individual meetings allow facilitators to meet participants and form a working relationship with them individually, thus helping all participants feel more comfortable in the group setting for the first session. They also allow facilitators to fully explain the program and answer any questions the participant may have that he or she may consider "silly" or inappropriate to ask in front of a group and allow full consenting of the individual privately. Individuals who consent to participate are enrolled in the program and remain in the same group throughout the program with the same facilitator for both group and individual sessions. After enrolling in the program, participants are asked to fill out a survey and asked to allow the facilitator to take height, weight, and saliva sample for baseline measurement of salivary cotinine. Participants are asked to let their group facilitator know what their choice of medication at the in-person session. Facilitators answer any questions the participant has and participants are given the option of having the study physician call them to discuss personal concerns. Estimated time for individual session is 50-60 minutes. Group facilitators call all participants 1-2 days prior to the group session as a reminder. All participants complete the medical screener for the appropriate medication. The study physician reviews all medical information prior to the first group session. Participants who do not meet medical eligibility for their choice of medication are called by the study physician to discuss options. Participants are reimbursed with a $20 gift card for their time.

#### Group Session 1

*The first group session is the quit date for all participants in the group*. By using one quit date, we ensure that participants are going through the quitting process together. As participants arrive, they are be asked to complete weekly surveys. Study medication is provided to participants at the group sessions, enough pills to last them until the next group session. The study physician is available to answer additional questions. Beginning with this session and continuing through all sessions, facilitators start the session with a prayer (if the group chooses to do so) and team building exercises. All discussions begin with each individual's discussion of his or her journey with tobacco and issues surrounding quitting. Individual facilitators decide how to ensure all participants who want to talk get a chance at each session. After personal discussion, the topic(s) for the session is discussed, using the associated educational materials (*see Table *2*for topics and educational materials*). Estimated time for the group session is 60-90 minutes. During the week between the group sessions, facilitators call participants to see how they are doing, provide counseling for personal issues, and to remind them of the next group session. Facilitators also ask about possible side effects of medication and any adverse events. Estimated time for each phone call is 15-20 minutes; though in our pilot groups some calls went as long as 30 minutes.

#### Group Sessions 2-7

Weekly session and telephone procedures follow the same format as for the first session. For discussion of traditional tobacco, participants are asked to bring any personal items surrounding the use of traditional tobacco. During that session, participants decide as a group if they would like to conduct a group ceremony signifying their journey with ending recreational use of tobacco and celebrating it as a sacred plant. Facilitators are sure to acknowledge the diversity of tobacco use (or nonuse) and adjust discussion and ceremony as appropriate. Participants watch the ANBL traditional tobacco video during the session and are asked to share personal stories if they choose.

#### Weeks 9-11

Participants receive one phone call per week from their facilitator. Calls follow the same procedures as those made in between group sessions.

#### Group Session (Week 12): End of Pharmacotherapy

Session discussion procedures follow the same format as for other group sessions. Participants who have remained abstinent provide saliva samples for cotinine analysis. Participants and facilitators bring food for a potluck dinner. Participants are reimbursed with a $20 gift card for their time. All participants, regardless of smoking status receive a certificate of achievement for completing the program. Estimated time is 60-90 minutes.

#### 6-Month Follow-Up

At 6-months post-baseline, participants are asked to return for a final group session. Participants self-reporting continuous abstinence are asked to participate in salivary cotinine analysis for verification. Participants are reimbursed with a $20 gift card for their time. Estimated time is 60-90 minutes.

#### 12-Month Follow-Up

At 12 months post-baseline, participants are asked to return for a final individual session. After initial assessment and collection of saliva sample (for those reporting continuous abstinence) and smoking status, they are asked to participate in a focus group about the program itself and how to improve it. Procedures for the focus group are discussed in *Process Measures and Evaluation*. Participants are reimbursed with a $20 gift card for their time. Estimated time is 30 minutes.

### Non-Tailored - Current Best Practices (CBP) Intervention Components

In this condition, non-Native facilitators follow a detailed script and use a guide (American Cancer Society) to provide education about the risks of smoking and how to quit. The initial telephone sessions reviews the health risks associated with smoking, the addictive nature of tobacco, and the disproportionate effects of tobacco. In addition, the facilitator and participant complete the exercises on making a plan to quit, which includes setting a quit date, and identifying a plan to use the pharmacotherapy. They also list smoking related habits and replacement activities. Towards the end of the session, participants are asked to restate what was agreed upon, and a verbal contract (i.e., pledge) is secured. The follow-up sessions include an assessment of progress towards meeting the quit goal, reviewing educational information, completing exercises from the guide (coping with urges; what to do in case of relapse, resetting goals, and securing additional contracts. Facilitators will be trained (and monitored for compliance) to avoid cultural-tailoring and maintaining protocol.

This counseling approach amounts to a fairly intensive education-based intervention. It incorporates the recommendations of current treatment guidelines which include giving clear advice to quit, providing assistance for quitting, and arranging follow-up. Health Education is designed to give participants additional information (such as health risks associated with smoking) and set specific goals related to smoking (e.g., a quit date). Health Education is characterized by providing information and advice, which results in the educator talking more than the participant. We also conduct on-going monitoring, using audiotapes of sessions, to prevent treatment contamination and to verify treatment fidelity.

Participants randomized to this arm are also all offered pharmacotherapy (e.g. varenicline or bupropion or NRT). In addition, they meet individually with a non-Native facilitator at weeks 1, 12 and 24 and 1-year for assessments, collection of saliva, and coordination of pharmacotherapy. Participants in this arm are provided 5 telephone sessions. These calls are delivered near their quit date and each individual call session is approximately 10 minutes.

Since this arm is not culturally-tailored, the self-help materials that are provided at the first individual session are non-tailored cessation information. Facilitators provide participants with a brief brochure of current practice guidelines for smoking cessation.

### Statistical Analyses/Sample Size and Power Calculations

#### Sample Size and Power

The primary endpoint is cotinine verified continuous abstinence at 12 months. We expect a 30% cessation rate in the **ANBL **group and a 15% cessation rate in the **CBP **group. Based on the two-sample test for the difference in proportions using un-pooled normal approximation adjusted for variance inflation due to intra-cluster correlation [35], along with the assumptions above and cluster size of 5 people and a very conservative estimate of intra cluster correlation coefficient of 0.1 due to lack of relevant data (hence inflation factor is 1.4), we will need 27 clusters in each arm to have 80% power to conclude the cessation rate of smokers who receive the ANBL counseling is higher than that of those who receive non-tailored counseling at 5% significance level (1-sided). To make the design balanced in both sites, we will have 14 clusters in each arm at each site, i.e. 28 clusters in each arm. Hence, we will need a total of 56 clusters (14 clusters/arm/site × 2 arms × 2 sites). Considering a conservative estimate of attrition rate of 30%, we propose to recruit 8 people for each cluster, resulting in a total of 56 × 8 = 448 AI/AN smokers.

### Statistical Analyses

#### Baseline Summary

We will first summarize all baseline characteristics globally and by treatment group. Categorical variables will be summarized by frequencies and percentages and quantitative variables will be summarized by means and standard deviations.

#### Hypothesis Testing

In order to take into account the intracluster correlation, the general estimation equations (GEE) will be applied to hypothesis testing on comparing the intervention effect at the individual-level for both the primary and secondary aims (see below) using SAS PROC GENMOD. We will examine correlation structures such as the exchangeable and the first-order autoregressive or the spatial-power, and determine the optimal structure based on the Akaike Information Criterion (AIC). In addition, for the exploratory purpose, we will also assess potential covariates and mediating factors including demographic, smoking history, psychological, and sociocultural factors that may contribute to smoking abstinence. Interaction effects among these factors will also be assessed.

#### Primary Aim

1. To test the efficacy of All Nations Breath of Life Program **(ANBL) **versus **CBP **on smoking cessation among AI/AN smokers.

We will compare the salivary cotinine verified continuous abstinence rates at 12 months (and at Week 12 in a secondary aim, see below) between the two groups using GEE. For our primary comparison, we will conduct comparisons using (i) only the participants who complete the study, (ii) all subjects by imputing the dropouts as smokers, and (iii) all subjects by multiple imputation assuming the dropout mechanism to be missing at random. In (iii), the imputation model will match the model for data analysis as suggested by Allison (2001).

To evaluate secondary endpoints, we will compare the salivary cotinine verified continuous abstinence rates between groups at Week 12 (end of pharmacotherapy). Finally, as we have no preliminary estimates of relapse based upon timeline follow-back, this will be an exploratory endpoint. We will compare the time to relapse between the two groups using the frailty models (the modified Cox regression models for correlated time to relapse) to adjust for intracluster correlation.

##### Cost-effectiveness analysis

The purpose of our cost-effectiveness analysis is to establish the cost-effectiveness of the ABNL program relative to standard care for smoking cessation. While our cost analytic framework follows the guidelines adopted by the CDC [36], we will assess costs from the service provider perspective rather than the recommended societal perspective. Given that there is widespread agreement that the cessation of smoking behavior confers important societal benefits valued far in excess of the costs needed to produce them, the research expenses needed to fulfill the societal perspective would exceed the benefit of that approach. Variable costs for each study arm will be valued in the year during which they are incurred. Discounting is not required for the six month intervention period, and research costs ($20 gift cards) will be excluded from analyses.

Program costs that will vary depending on the number of participants include personnel time, phone charges, materials (brochures and other handouts), pharmacotherapy, and participant incentives. Facilitator time for group sessions and individual telephone counseling will be documented using a time diary/log and valued at wage rates including benefits. Phone charges for local and long distance calls will be applied to the time for telephone-based counseling. Costs to produce brochures and other handouts will be tracked as they are printed. Pharmacotherapy costs will be valued according to IHS medication costs inclusive of dispensing fees. For sensitivity analyses, we will vary pharmacotherapy costs according to prevailing retail rates (market value) using the average of no fewer than three price sources (local pharmacy as well as on-line pharmacy options). Mailing costs will be added if appropriate. In addition, time for physician prescribing and medication counseling as well as pharmacist counseling will be included for the project physician and project pharmacist (valued at wages plus benefits). Items included as incentives (quit kits, stress balls, photo magnets, water bottles, pens, bracelets, t-shirts) will be valued at their retail prices. Videos and CDs each cost $1 under the KUMC license, and the Rez-robics DVDs will not be included as they are available for free.

#### Incremental Cost Analyses

We will report an incremental cost-effectiveness ratio for the two arms. Incremental cost analyses identify the marginal benefit of switching from one intervention to the other relative to the increased cost. Since we hypothesize that ABNL will be more effective and more costly, the incremental cost-effectiveness analysis will inform us as to whether the additional costs associated with ABNL produce an acceptable level of benefit.

The effectiveness measure is the biochemically-verified, 7-day quit rate at 12 months.

In the event that pharmacotherapy costs make CBP more expensive than ABNL, then the ICER will be adjusted according with consideration of relative effectiveness.

#### Sensitivity Analyses

All cost analyses invariably require certain assumptions. The data derived for this cost analysis come from a clinical trial based in Kansas and Oklahoma, so extrapolation of the cost analysis requires adjustment of wage rates. For instance, health care related wages in Kansas are 86.0% of the national average [37]. Therefore, in sensitivity analyses, we will adjust wages rates upwards to a national average.

Likewise, the generalizability of a single clinical trial result to other populations necessitates that we explore how the variation in counseling time and effectiveness influence the relative cost-effectiveness of the treatment strategies. In the sensitivity analysis, we will vary these measures +/- 2 standard deviations.

## Summary

Currently, very few culturally-tailored smoking cessation studies are carried out in the American Indian population. The results from this randomized clinical trial will contribute to the limited information on effective smoking cessation programs for American Indian smokers. American Indians have the highest rates of smoking prevalence of any racial/ethnic group in the US, and hopefully, the findings from this study will ultimately help reduce the smoking-related health disparities in this underserved population.

## Abbreviations

AI: American Indian; AIC: Akaike Information Criterion; AN: Alaska Native; CAB: Community Advisory Board; CBP: Current Best Practices; CD: Compact Disc; CDC: Center for Disease Control; DHS: Doctors Helping Smokers (a study acronym); DVD: Digital Versatile Disc; GAINS: Giving American Indians No-Smoking Strategies study acronym); GEE: Generalized Estimation Equation (a statistical method); IHS: Indian Health Services; KUMC: University of Kansas Medical Center; MI: Motivational Interviewing; NRT: Nicotine Replacement Therapy; SAS PROC GENMOD: a statistical procedure in SAS;

## Competing interests

The authors declare that they have no competing interests.

## Authors' contributions

WSC, BF, LAB, KAG, TS, CMD participated in the design of the study; HY led the statistical and power calculations; MT and LC are participating in the development and implementation of the intervention; BK is coordinating the study. All the authors have read, revised and approved the final manuscript.
